# Dr. Smee’s Case of Violent Hysteria in a Man

**Published:** 1842-08-27

**Authors:** Alfred Smee


*Violent Hysteria in a Man.* By ALFRED SMEE, Esq., F. R. S.—This was a well marked case, characterized by the most convulsive laughter, crying, &c. His strength was such that it required seven or eight men to hold him. There was great heat over the parietal bones, over which cold water was freely applied; and the heat on this region of the skull was generated so fast, that the cold water evaporated as if thrown on a hot substance, and rose in vapour. The man's age was twenty-six; he was small, weak, and effeminate, of an excitable temperament. It is not stated, as it ought to have been, whether he be married or not.—*Ibid, from Medical Gazette*, No. xxvii, March 25, 1842.

[Another and still more remarkable case is given in the 33d No. of the same Journal, (May 6, 1842,) by Mr. Stanger, of Nottingham. In this case the hysteric convulsions were controlled, and a cure effected, by the threat of a red hot poker being applied to a particular part of the back. The patient was a robust-looking man, in his eighteenth year.]—*Rev.*

